# Problematic Use of the Internet Mediates the Association between Reduced Mentalization and Suicidal Ideation: A Cross-Sectional Study in Young Adults

**DOI:** 10.3390/healthcare10050948

**Published:** 2022-05-20

**Authors:** Francesco Saverio Bersani, Tommaso Accinni, Giuseppe Alessio Carbone, Ornella Corazza, Angelo Panno, Elisabeth Prevete, Laura Bernabei, Chiara Massullo, Julius Burkauskas, Lorenzo Tarsitani, Massimo Pasquini, Massimo Biondi, Benedetto Farina, Claudio Imperatori

**Affiliations:** 1 Department of Human Neurosciences, Sapienza University of Rome, 00185 Rome, Italy; tommaso.accinni@uniroma1.it (T.A.); elisabeth.prevete@uniroma1.it (E.P.); laura.bernabei@uniroma1.it (L.B.); lorenzo.tarsitani@uniroma1.it (L.T.); massimo.pasquini@uniroma1.it (M.P.); massimo.biondi@uniroma1.it (M.B.); 2 Cognitive and Clinical Psychology Laboratory, Department of Human Sciences, European University of Rome, 00163 Rome, Italy; giuseppea.carbone@gmail.com (G.A.C.); angelo.panno@unier.it (A.P.); benedetto.farina@unier.it (B.F.); claudio.imperatori@unier.it (C.I.); 3 Department of Clinical, Pharmaceutical and Biological Sciences, University of Hertfordshire, Hatfield AL10 9EU, UK; o.corazza@herts.ac.uk; 4 Mental Health Department, ASL Roma 5 Hospital, 00184 Rome, Italy; 5 Experimental Psychology Laboratory, Department of Education, Roma Tre University, 00185 Rome, Italy; chiara_massullo@yahoo.it; 6 Laboratory of Behavioral Medicine, Neuroscience Institute, Lithuanian University of Health Sciences, 00135 Palanga, Lithuania; julius.burkauskas@lsmuni.lt

**Keywords:** problematic use of internet, social media addiction, internet gaming disorder, suicidal behavior, mentalization, psychopathology

## Abstract

Suicide is a major public health problem, and it is urgent to investigate its underlying clinical and psychological concomitants. It has been suggested that low mentalization skills and problematic use of the internet (PUI) are factors that can play a role in suicidal behaviors. It is possible that poor mentalization skills contribute to leading to forms of PUI, which, in turn, can represent triggers for suicidal ideation (SI). We tested this hypothesis through a quantitative and cross-sectional study on a sample (n = 623) of young adults (age range: 18–34). Self-report measures investigating symptoms related to Social Media Addiction (SMA), Internet Gaming Disorder (IGD), mentalization capacity, and SI were used. A single mediation analysis with two mediators was carried out to evaluate the direct and indirect effects of mentalization on SI through the mediating role of SMA- and IGD-related symptoms, controlling for potential confounding factors (e.g., socio-demographic and addiction-related variables). The four explored variables were significantly associated with each other (all *p* < 0.001) across all subjects; the mediational model showed that the total effect of mentalization on SI was significant (B = −0.821, SE = 0.092 (95% CI: −1.001; −0.641)) and that both SMA- (B = −0.073, SE = 0.034 (95% CI: −0.145; −0.008)) and IGD-related symptoms (B = 0.046, SE = 0.027 (95% CI: −0.107; −0.001)) were significant mediators of such association. Our findings support the possibility that PUI severity plays a relevant role in mediating the association between low mentalization skills and levels of SI.

## 1. Introduction

Suicide is a major public health problem. According to a recent World Health Organization report, it causes over 700,000 deaths per year globally, with an age-standardized suicide rate of 9 per 100,000 population and higher suicide rates in males than in females [1]. Suicide is an especially relevant issue in relation to adolescents and young adults, representing one of the leading causes of death for people aged between 15 and 29 [1]. As a consequence, although certain suicide-related risk factors (e.g., mental illness, previous suicide attempts, fragile socio-economic status, discrimination, disabling physical diseases, harmful use of alcohol) and protective factors (e.g., strong personal relationships and positive coping) have been identified [2], it is urgent to further investigate the underlying clinical and psychological elements involved in suicidal behaviors (which have been defined as “*a range of behaviours that include thinking about suicide (or ideation),* *planning for suicide, attempting suicide and suicide itself*” [2]) in young individuals.

It has been suggested that difficulties in emotion regulation among young people are related to suicide. Theories, in fact, have postulated that suicidal behaviors can serve as means to escape from aversive self-awareness [3] and that they are forms of maladaptive responses to stressful situations characterized by the presence of feelings of defeat and of the perception of no escape and no rescue [4,5]. Further, certain psychiatric disorders showing marked emotion dysregulation such as Bipolar Disorder and Borderline Personality Disorder are associated with increased suicide rates [6,7]. Mentalization, an evolutionarily prewired capacity defined as “*the capacity to understand one’s own and others’ internal mental processes, such as thoughts, feelings, needs, desires, and motivations, and their relationship to behavior*” [8], has been proposed to be closely related to emotion regulation, and low mentalization capacity has preliminarily been linked to suicidal behaviors [9,10,11,12,13]. Difficulties in mentalizing internal, interpersonal, social, and external stimuli, in fact, can lead to emotional dysregulation, which, in turn, can represent a trigger for suicidal behaviors.

An additional factor involved in suicidal behaviors among young individuals is the exposure to dysfunctional forms of internet use. Such phenomenon is often referred to as problematic use of internet (PUI), and it has been considered a form of behavioral addiction [14,15,16,17]. PUI can contribute to the emergence of different psychopathological symptoms: it has been associated with mood, anxiety, and eating disturbances, as well as to addiction and to functional impairments [15,18,19,20]. Suicidal behavior is a worrying concomitant of PUI: as evidenced by a recent meta-analysis of cross-sectional studies [21], individuals with PUI show increased rates of suicidal ideation (SI), planning, and attempts.

While the association between PUI and forms of suicidal behavior has been observed in young individuals, the mechanisms underlying such link have not been clarified. It has been hypothesized that phenomena of “deindividuation” caused by overuse of the internet, access to unsafe virtual environments, and shared vulnerability factors between the two phenomena can represent causal factors linking PUI with suicidal behaviors (as discussed in [22]), although research in the field is still in a preliminary phase.

The difficulties in elucidating the underlying mechanisms are further increased by the fact that the construct of PUI can involve several online activities that vary from each other and only partially overlap in terms of features, concomitants, and manifestations, such as gaming, buying, social networking, gambling, and using pornography [15,23,24]. Certain facets of PUI are increasingly reaching the status of clinically relevant disturbances, especially in relation to young people. Specifically, Internet Gaming Disorder (IGD), i.e., a problematic form of online videogame (OVG) use, is listed as a potential independent nosologic diagnosis within the *Conditions for Further Study* section of the latest edition of *The Diagnostic and Statistical Manual of Mental Disorders* (DSM-5) [25], while the diagnosis of Gaming Disorder has been included in the latest edition of *The International Classification of Diseases* [26]. Similarly, the condition of Social Media Addiction (SMA) has been investigated as a form of dysfunctional use of social media (SM) characterized by addiction-related symptoms (e.g., tolerance, craving, and abstinence) and impaired functioning [27,28,29].

Preliminary studies have documented a relationship between dysfunctional use of SM or OVG, i.e., two common forms of PUI among young adults, and forms of suicidal behaviors [30,31,32,33,34,35]. Of relevance, certain forms of behavioral addictions (as well as of substance addictions) have been associated with lower mentalization capacity [36,37,38,39,40,41,42,43]; in fact, emotional dysregulation and difficulties in mentalizing internal/external stimuli can have a role in triggering addictive behaviors. Subsequently, it is possible that one way by which reduced mentalization capacity favors suicidal behaviors in young people is through the occurrence of phenomena related to PUI: poor mentalization skills may contribute to leading to forms of PUI, which, in turn, may represent additional triggers for SI.

To summarize, theoretical considerations suggest that lower mentalization capacity can lead to increased emotional dysregulation, and that increased emotional dysregulation can favor the development of various psychopathological disturbances including suicidal and addictive behaviors. Based (i) on such considerations, (ii) on the mentioned preliminary evidence linking lower mentalization capacity with suicidal behaviors and addictive behaviors, and (iii) on the mentioned preliminary evidence linking PUI with suicidal behaviors, in the present study, we aimed to cross-sectionally explore this phenomenon. Specifically, we aimed to elucidate whether lower mentalization capacity is associated with higher levels of SI in a sample of young adults (age: 18–34), and whether the severity of symptoms related to certain aspects of PUI (i.e., SMA- and IGD-related symptoms) has a role in mediating such association.

## 2. Materials and Methods

### 2.1. Participants

According to the sample size guidelines of Fritz and MacKinnon [44], in the mediation model, assuming small effect size for both “a” (i.e., the effect of the independent variable on the mediator) and “b” (i.e., the effect of the mediator on the dependent variable controlled for the independent variable) paths, the analyses required a minimum sample size of 558 with the bootstrapping procedure to provide a statistical power of 0.80.

The sample of the study consisted of 623 Italian young adults (449 females, 174 males; mean age: 24.40 ± 3.72 years; age range: 18–34). Young adults were recruited as studies suggest that PUI and suicidal behaviors are especially worrying phenomena among subjects in this age range [2,45], potentially due to a range of circumstances including a higher prevalence of psychopathological disturbances and developmental challenges related to the transition to adulthood. Recruitment was performed between September 2020 and March 2021 through an online survey shared using web-based tools (e.g., emails, SM, and instant messaging). The inclusion criteria were (i) age between 18 and 34 years, (ii) good ability to understand Italian, (iii) correct response to one item of an attentional quality check (e.g., “*Please score answer #5*”), and (iv) provision of consent within the survey.

Participants voluntarily and anonymously responded to the survey. They did not receive payment or other compensations for their participation in the study. Ethics approval was obtained from the Institutional Board of the Department of Human Neurosciences of Sapienza University of Rome. This study is part of a larger project on the relationship between psychopathology and behavioral addictions; thus, the included sample partially overlaps with the sample presented in articles previously published by our research group [46,47].

### 2.2. Measures

Socio-demographic data of the recruited individuals were collected (age, sex, employment, educational achievements, substances or tobacco use, and body mass index (BMI)). Participants were asked to compile the following questionnaires: *Mentalization Questionnaire* (MZQ) [48], *The Bergen Social Media Addiction Scale* (BSMAS) [27], two items of the *Brief Symptoms Inventory* (BSI) investigating SI [49], *The Cut–Annoyed–Guilty–Eye Questionnaire* (CAGE) [50], and the *Internet Gaming Disorder Scale*—*Short Form* (IGDS9-SF) [51].

The MZQ is a 15-item self-administered scale based on the mentalization theory measuring the capacity to represent and understand inner mental states in oneself and others. Respondents rate the items (e.g., “*Most of the time I don’t feel like talking about my thoughts and feelings with others*”, “*Sometimes feelings are dangerous for me*”) on a five-point Likert scale (from 1 = completely disagree to 5 = completely agree); higher scores reflect better mentalization skills [48]. In the original validation study [48], satisfactory psychometric properties (e.g., adequate internal consistency) were reported. In the present study, the Italian version of the MZQ was used [39,40], and the Cronbach’s α was 0.85 for the total score.

The BSMAS is a reworking of the Bergen Facebook Addiction Scale and contains six items reflecting addiction-like symptoms (i.e., salience, mood modification, tolerance, withdrawal, conflict, and relapse) related to the dysfunctional use of SM during the previous year [27]. Each item (e.g., “*How often during the last year have you used social media so much that it has had a negative impact on your job/studies*?”, “*How often during the last year have you felt an urge to use social media more and more*?”) is answered on a five-point Likert scale (from 1 = very rarely to 5 = very often). A cut score of ≥19 has previously been employed in ascertaining SMA [28,46,47,52]. In the present study, the Italian version of the BSMAS was used [29], and the Cronbach’s α was 0.84.

The IGDS9-SF is a tool used to assess IGD-related symptoms [51,53]. The IGDS9-SF includes nine items exploring disturbances related to the IGD criteria proposed in the DSM-5. All nine items (e.g., “*Do you systematically fail when trying to control or cease your gaming activity*?”, “*Do you feel more irritability, anxiety or even sadness when you try to either reduce or stop your gaming activity*?”) are focused on disturbances occurring during the previous year and are answered on a five-point Likert scale (from 1 = never to 5 = very often). Higher scores suggest higher levels of IGD-related disturbances. In the present study, the Italian version of IGDS9-SF [46,54] was used. The cut score of 21 has been suggested to identify individuals who present significant symptoms of IGD in an Italian-speaking sample [54]. In the current sample, the Cronbach’s α was 0.87.

Consistent with previous studies [55,56,57], in the current report, we used the sum score of items 9 (“*over the last week how much you suffered from thoughts of ending your life*”) and 39 (“*over the last week how much you suffered from thoughts of death or dying*”) of the BSI scale, i.e. a self-report inventory designed to investigate general psychopathology during the previous week [49], in order to assess SI (BSI-SI). Items are rated on a four-point Likert scale (from 0 = not at all to 4 = extremely), with higher values indicating higher severity of symptoms. SI was considered to be present if there was a score of 4 or more on the two items [55,56,57]. The Italian validated version used in this study showed good psychometric properties [58]. The Cronbach’s α of BSI-SI in our sample was 0.81, and the correlation between the two items was significant (rho = 0.675; *p* < 0.001).

The CAGE is a questionnaire commonly used to detect problematic alcohol use (PAU) based on four dichotomous items (e.g., “*Have you ever felt you should cut down on your drinking*?”, “*Have people annoyed you by criticizing your drinking*?”). Higher values indicate more severe problems related to alcohol use. A score ≥ 2 is often used for detecting PAU [40,59]. In the present study we used the Italian adaptation of the CAGE [60], and the Cronbach’s α was 0.68.

### 2.3. Statistical Analyses

All the analyses were performed using the SPSS (26.0) statistical package (IBM, Armonk, NY, USA). The relationships among the study variables were assessed using Spearman’s rho correlation coefficients.

To determine whether the relationship between mentalization capability and BSI-SI was mediated by both SMA and IGD-related symptoms, a single mediation analysis with two mediators was run using the “Model 4” option of the SPSS Macro Process [61] with 5000 bootstrap samples. Specifically, the MZQ total score (i.e., mentalization capacity) was the independent variable; the BSI-SI total score was the dependent variable; and both the BSMAS total score (i.e., SMA-related symptoms) and IGDS9-SF total score (i.e., IGD-related symptom) were the mediators. In this model, sociodemographic (i.e., sex, age, educational level, occupation, and marital status) and addiction-related (i.e., use of alcohol, tobacco, and substances) variables were included as covariates in order to adjust for potential confounding factors. BMI was also included as a covariate due to its known relationship with PUI [46,47,62,63].

According to Baron and Kenny [64], we reported three separate pathways. In the first pathway (pathway a), we showed the direct effect of mentalization capability on SMA-/IGD-related symptoms. In the second pathway (pathway c′), we reported the direct effect of mentalization capability on SI. In the third and last pathway (pathway c), we showed the total effect, i.e., the sum of direct and indirect effects, of MZQ on BSI-SI. Pathway b (i.e., the association of SMA- and IGD-related symptoms with the BSI-SI total score) and pathway ab (i.e., the indirect effect of mentalization levels on BSI-SI through SMA- and IGD-related symptoms) are also reported.

Although the relationships between the associated variables are interpreted as causal in mediational models, in cross-sectional studies, such models should be viewed as a type of variance partitioning, which can be useful in elucidating whether the relationship between two variables is reduced or increased when a mediation variable is taken into consideration [65]. Thus, it should be noted that the statistical design of the current research is correlational, and, accordingly, it does not allow one to establish unequivocal interpretations of causal effects.

## 3. Results

The descriptive statistics are reported in Table 1. In the present sample, according to the cut scores described above, there were 177 individuals (28.4%) who presented SMA, 37 (5.9%) who presented IGD, 77 (12.4%) who presented SI, and 80 (12.8%) who presented PAU.

Our results showed that the MZQ total score was significantly and negatively correlated with the IGDS-SF (rho = −0.196; *p* < 0.001), BSMAS (rho = −0.338; *p* < 0.001), BSI-SI (rho = −0.342; *p* < 0.001), and CAGE total scores (rho = −0.159; *p* < 0.001). The MZQ total score was also significantly and positively correlated with age (rho = 0.090; *p* = 0.024). BSI-SI was significantly negatively correlated with age (rho = −0.103; *p* = 0.010) and significantly positively associated with CAGE (rho = 0.095; *p* = 0.018) and both the BSMAS (rho = 0.260; *p* < 0.001) and IGDS-SF (rho = 0.132; *p* < 0.001) total scores. Although similar patterns of correlations were observed for both the BSMAS and IGDS-SF total scores, only IGD-related symptoms were significantly associated with self-reported BMI (rho = −0.090; *p* < 0.05) and only SMA-related symptoms were significantly associated with the CAGE total score (rho = 0.100; *p* = 0.013) and with age (rho = −0.192, *p* < 0.001). Detailed correlations are shown in Table 2.

**Figure 1:**
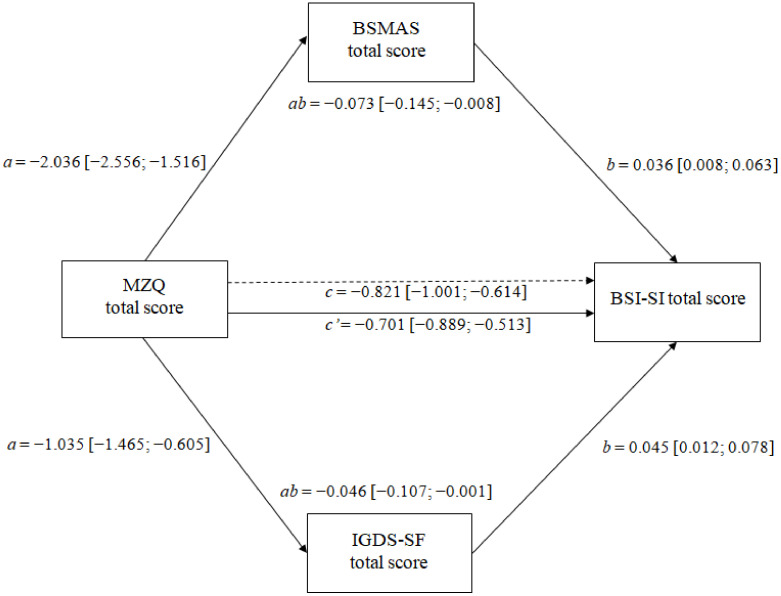
Graphical representation of the results from the mediation model. The reported estimates were obtained controlling for potentially competing factors (i.e., socio-demographic variables, tobacco use, problematic alcohol use, drug use, and Body Mass Index). Abbreviations: MZQ = Mentalization Questionnaire; BSMAS = Bergen Social Media Addiction Scale; IGDS-SF = Internet Gaming Disorder Scale–Short-Form; CAGE = Cut–Annoyed–Guilty–Eye (CAGE) Questionnaire; BMI = Body Mass Index; BSI-SI= Brief Symptom Inventory-Suicidal Ideation.

The results of the mediational model are graphically shown in Figure 1 (and fully reported in Supplementary Table S1). The total effect was negative and significant (B = −0.821, SE = 0.092 (95% CI: −1.001; −0.641)), indicating that lower mentalization capability was associated with a higher BSI-SI. Moreover, the effect of mentalization capability on both SMA- (B = 2.036, SE = 0.265 (95% CI: −2.556; −1.516)) and IGD-related symptoms) B = −1.035, SE = 0.219 (95% CI: −1.465; −0.605)) was negative and significant. In turn, both SMA- (B = 0.036, SE = 0.096 (95% CI: 0.008; 0.063)) and IGD-related symptoms) B = 0.045, SE = 0.017 (95% CI: 0.012; 0.078)) were positively associated with BSI-SI. The direct effect of mentalization capability on BSI-SI was negative and significant (B = −0.701, SE = 0.096 (95% CI: −0.889; −0.513)). Lastly, the indirect effect was significant for both the BSMAS (B = −0.073, SE = 0.034 (95% CI: −0.145; −0.008)) and IGDS-SF total scores (B = 0.046, SE = 0.027 (95% CI: −0.107; −0.001)), suggesting that the association between mentalization capability and SI was significantly mediated by both SMA- and IGD-related symptoms. As an exploratory analysis, we also tested whether the paths of the model (Figure 1) varied as a function of the sex and age of the participants. No evidence for a significant interaction emerged for sex (all *p* > 0.093). The only significant moderation effect observed for age was the interaction of MZQ X Age with BSI-SI (B = 0.072, SE = 0.023 (95% CI: 0.026; 0.118)). A simple slope analysis revealed that at lower ages, the association between MZQ and BSI-SI increased (B = −1.096, SE = 0.127 (95% CI: −1.346; −0.846)), even though it remained significant for the mean (B = −0.827, SE = 0.091 (95% CI: −1.006; −0.649)) and higher levels of age (B = −0.559, SE = 0.124 (95% CI: −0.803; −0.315)).

## 4. Discussion

Based on the preliminary evidence linking lower mentalization capacity with suicidal behaviors [9,10,11,12,13] and addictive behaviors [36,37,38,39,40,41,42,43], and linking PUI with suicidal behaviors [21,30,31,32,33,34,35], in the present study, we aimed to cross-sectionally explore this phenomenon and to elucidate, in a sample of 623 young adults, (i) whether lower mentalization capacity is associated with higher levels of SI, and (ii) whether the severity of symptoms related to certain aspects of PUI has a role in mediating such association. The results of the study highlighted that lower mentalization skills were significantly associated with higher levels of SI, and that such relationship was significantly mediated by SMA- and IGD-related symptoms, with relevant confounding factors (including age, sex, student status, educational level, BMI, use of tobacco, alcohol, and substances) being controlled for.

These results raise the possibility that one way by which reduced mentalization skills favor SI is through the occurrence of the maladaptive use of SM and OVG. Several mechanisms linking low mentalization capacity and dysfunctional SM/OVG use with suicidal behaviors have been proposed, which include complex interactions between biological, social, and psychological phenomena.

Previous data have suggested that impairments in mentalization capacity can have a role in favoring psychopathological conditions involving alexithymia, difficulties in emotional recognition, and negative affect [41,66,67], as well as behavioral or substance addictions [36,37,38,39,40,41,42,43]. Such data suggest that low levels of mentalization and emotional regulation are related to increased vulnerability for a wide range of dysfunctional emotions, thoughts, and behaviors, which, in turn, can favor the occurrence of SI. On the other hand, adequate mentalization abilities have been observed to have adaptive effects on mental health [68,69]. Studies have also observed that certain brain networks involved in mentalization processes [67,70] can be altered in certain psychopathological processes, possibly further underlining the relationships between mentalization and psychopathology.

The findings of our study add to this field of research by showing a potential role of dysfunctional SM and OVG use in the link between low mentalization capacity and SI. The findings are consistent with the previously proposed concept that mentalization deficits and the inability to contain emotions contribute to an inclination for addictive behaviors under emotional stress [36,37,38,39,40,41,42,43], and are consistent with the observation that addiction-related symptoms (e.g., mood alterations, impulsivity, and withdrawal symptoms related to both substances and certain behavioral addictions) can increase vulnerability to SI [21,22,30,31,32,34,35,71,72,73,74,75].

Of relevance, it is possible that PUI also favors suicidal behaviors through mechanisms that are independent from the condition of addiction per se: (i) emulation plays a key role in promoting suicidal behaviors, as documented by the research on the so-called Werther Effect, and both SM and OVG can expose users to unsafe virtual environments with subsequent increased risk of imitation (e.g., the contagion effect and cybersuicide pacts) [76,77,78,79,80,81]; (ii) overuse of the internet can be associated with increased symptoms of dissociation [82,83,84], which may result in increased suicide risk [85], as well as with phenomena of deindividuation [22]; (iii) studies have suggested that excessive violent game play can be related to certain factors linked with suicidal behaviors, such as acquired capability for suicide, fearlessness of death, and pain tolerance [33,86,87]; (iv) PUI appears to promote or worsen psychopathological conditions such as irritability and insomnia [46,88,89,90,91,92,93,94], thus contributing to worsening individuals’ psychopathological states; and (v) victims of cyberbullying have been observed to be at increased risk of self-harm and suicidal behaviors [95].

Overall, as mentalizing involves the capacity of understanding one’s own and others’ mental processes, it is possible that not only reduced mentalization skills favor SI through increased vulnerability to the development of PUI, but also that those (above-mentioned) features of PUI that can *per se* be related to increased suicidal behaviors are experienced as amplified in individuals with pre-existing low mentalization skills.

The reported data have potential clinical implications. According to the described cut scores of the BSI-SI scale, in the present sample, 77 subjects (12.4%) showed SI; such value is not far from the 12-month SI prevalence of 10.6% observed in a recent meta-analysis including 634,662 college students [96], although prevalence estimates of SI in young adults vary widely across different studies [97,98,99]. Further, according to the described cut scores of the BSMAS, IGDS9-SF, and CAGE scales, in the present sample, there were 177 subjects (28.4%) showing SMA, 37 subjects (5.9%) showing IGD, and 80 subjects (12.8%) showing PAU. Such rates of SI and of conditions that can contribute to increasing the risk of suicidal behaviors underline the relevance of implementing comprehensive programs to address the suicide risk in young adults. The evaluation of the presence/severity of certain forms of PUI in individuals with SI can allow healthcare professionals to identify behaviors that can have a role in the maintenance of SI.

Of relevance, while the present study is focused on the dysfunctional use of SM and OVG and on their implication in SI and psychopathological phenomena, other researchers have suggested that such internet activities can, in certain circumstances, contribute to facilitating sociality, peer support, and access to care or to suicide prevention programs [79,100,101]. Thus, the findings of the current study do not apply to SM and OVG use *per se*, but rather to the symptoms related to IGD and SMA, i.e., dysfunctional forms of SM and OVG use; relatedly, it should also be remarked that “studies suggest that gaming disorder affects only a small proportion of people who engage in digital- or video-gaming activities” [102].

Several limitations should be considered in relation to the current research. We used only self-report measures, which are known to be affected by several biases. Our study used a cross-sectional design, but in order to infer clear causal relationship between correlated variables, a longitudinal approached should be implemented. We focused on a sample of young adults recruited from a general population sample; thus, data on the participants’ psychiatric history were not collected. It is possible that the severity of the clinical variables investigated in the current study is influenced by the fact that all the participants were recruited after the COVID-19 outbreak, which is a risk factor for psychopathology, and also by the fact that the participants were recruited online (this may have facilitated the inclusion of subjects with higher exposure to internet activities). Certain groups of individuals are more frequent in the enrolled sample (e.g., females compared to males, students compared to non-students). Lastly, we assessed SI using two items of the BSI scale, i.e., a self-report inventory designed to investigate general psychopathology; while this approach has already been previously used in the literature [55,56,57], more comprehensive scales by which to assess SI and suicidal behavior exist, whose use may lead to an increased accuracy of the findings. Among the strengths of the study: (i) this is, to the best of our knowledge, the first research specifically aimed at investigating the mediating role of SMA- and IGD-related symptoms on the relationship between mentalization and SI in young adults; (ii) the sample size (n = 623) was adequate, as determined by *a priori* power analyses; (iii) we used extensively validated assessment tools, which showed satisfactory reliability in the enrolled sample; (iv) we statistically adjusted for certain potential confounders.

## 5. Conclusions

In conclusion, our findings contribute to elucidate the complex relationships between SI, mentalization capacity, and symptoms related to SMA and IGD. It is possible that the interaction between psychological disturbances related to low mentalization capacity and to the dysfunctional use of SM and OVG can expose young people to increased SI as well as to other psychopathological disturbances. Given the risks related to suicide for young adults and the diffusion of SM and OVG use among young people, specific attention should be paid to these phenomena by researchers, policymakers, and clinicians.

## Figures and Tables

**Table 1:** Descriptive statistics for the sample (N = 623).

Variables	
Age—M ± SD	24.40 ± 3.73
Females—N (%)	449 (72.1)
Employed—N (%)	179 (28.7)
Unemployed—N (%)	40 (6.4)
Students—N (%)	404 (64.8)
Married or living with partner—N (%)	73 (11.7)
Educational level > 13 years—N (%)	261 (41.9)
Tobacco use in the last 12 months—N (%)	226 (36.3)
Substance use in the last 12 months—N (%) *	73 (11.7)
CAGE total score—M ± SD	46 ± 0.90
CAGE ≥ 2—N (%)	80 (12.8)
BMI—M ± SD **	22.19 ± 3.40
MZQ total score—M ± SD	3.18 ± 0.79
BSMAS total score—M ± SD	15.49 ± 5.53
BSMAS ≥ 19—N (%)	177 (28.4)
IGDS-SF—M ± SD	11.71 ± 4.53
IGDS-SF ≥ 21—N (%)	37 (5.9)
BSI-suicidal ideation—M ± SD	1.01 ± 1.89
BSI-suicidal ideation ≥ 4—N (%)	77 (12.4)

Abbreviations: M = mean; SD = standard deviation; CAGE = Cut–Annoyed–Guilty–Eye (CAGE) Questionnaire; BMI = Body Mass Index; MZQ = Mentalization Questionnaire; BSMAS = Bergen Social Media Addiction Scale; IGDS-SF = Internet Gaming Disorder Scale–Short-Form; BSI = Brief Symptom Inventory. * Number of individuals who reported that the most frequently used psychoactive substance in the previous year was one of the following: cannabis, cocaine, heroin or other opiates, hallucinogens, amphetamines or other psychostimulants, tranquillizers, other substances different from alcohol, nicotine, caffeine, and hyper-caloric food. ** Based on self-reported height and weight.

**Table 2:** Associations between variables in the sample (N = 623).

	1	2	3	4	5	6
1. MZQ total score	-					
2. BSMAS total score	−0.338 ***	-				
3. IGD-SF total score	−0.196 ***	0.165 ***	-			
4. BSI-suicidal ideation	−0.342 ***	0.260 ***	0.132 ***	-		
5. CAGE total score	−0.159 ***	0.100 *	0.059	0.095 *	-	
6. Age	0.090 *	−0.192 ***	−0.065	−0.103 **	0.044	-
7. Self-reported BMI	0.010	−0.051	0.113 **	−0.046	0.082 *	0.141 ***

Abbreviations: MZQ = Mentalization Questionnaire; BSMAS = Bergen Social Media Addiction Scale; IGDS-SF = Internet Gaming Disorder Scale–Short-Form; CAGE = Cut–Annoyed–Guilty–Eye (CAGE) Questionnaire; BMI = Body Mass Index; BSI= Brief Symptom Inventory. Note: * *p* < 0.05; ** *p* < 0.01; *** *p* < 0.001.

## Data Availability

Aggregated data may be available from the corresponding author upon reasonable request.

## Supplementary Materials

The following supporting information can be downloaded at: https://www.mdpi.com/article/10.3390/healthcare10050948/s1, Table S1: Results of the mediation model with the BSMAS and IGDS total score as mediators.
