# Supplementary material for: Problematic Use of the Internet Mediates the Association between Reduced Mentalization and Suicidal Ideation: A Cross-Sectional Study in Young Adults
Source: Healthcare (Basel). 2022 May 20;10(5):948. doi: 10.3390/healthcare10050948 (PMC9140488; doi:10.3390/healthcare10050948)
Supplement: Supplementary file 1 [file healthcare-10-00948-s001.zip › healthcare-1666579-supplementary.pdf]

Supplementary Table S1. Results of the mediation model with the BSMAS and IGDS total score as mediators.

	Pathway *a*						Pathway *c'*			Pathway *c*		
	BSMAS total score			*IGDS total score*			*BSI-suicidal ideation total score*					
	B	SE	95% CI	*B*	SE	95% CI	*B*	SE	95% CI	*B*	SE	95% CI
MZQ total score	**−2.036*****	0.265	[−2.556; −1.516]	**−1.035*****	0.219	[−1.465; −0.605]	**−0.701*****	0.096	[−0.889; −0.513]	**−0.821*****	0.092	[−1.001; −0.641]
BSMAS total score	-	-	-	-	-	-	**0.036****	0.014	[0.008; 0.063]	-	-	-
IGDS total score	-	-	-	-	-	-	**0.45****	0.017	[0.012; 0.078]	-	-	-
Age	−0.093	0.070	[−0.231; 0.045]	−0.025	0.058	[−0.139; 0.090]	−0.028	0.024	[−0.075; 0.019]	−0.032	0.024	[−0.080; 0.016]
Gender	**1.643*****	0.476	[0.708; 2.579]	**−3.368*****	0.394	[−4.141; −2.594]	0.082	0.176	[−0.263; 0.426]	−0.011	0.165	[−0.335; 0.313]
Occupation	**−1.481*****	0.426	[−2.318; −0.643]	−0.623	0.352	[−1.315; 0.069]	0.061	0.147	[−0.229; 0.351]	−0.020	0.148	[−0.310; 0.313]
Educational level	0.232	0.425	[−0.603; 1.067]	−0.314	0.351	[−1.004; 0.377]	−0.022	0.146	[−0.308; 0.264]	−0.028	0.147	[−0.317; 0.261]
Marital status	−0.903	0.689	[−2.256; 0.450]	−0.131	0.570	[−0.988; 1.250]	−0.215	0.236	[−0.679; 0.249]	−0.241	0.239	[−0.710; 0.227]
Self-reported BMI	0.042	0.062	[−0.081; 0.164]	0.048	0.052	[−0.053; 0.149]	−0.013	0.021	[−0.055; 0.029]	−0.010	0.022	[−0.052; 0.033]
Tobacco use	−0.446	0.432	[−1.295; 0.403]	−0.659	0.357	[−1.361; 0.043]	−0.229	0.148	[−0.521; 0.062]	−0.275	0.150	[−0.569; 0.019]
CAGE ≥ 2	0.986	0.629	[−0.248; 2.221]	0.084	0.520	[−0.937; 1.105]	0.141	0.216	[−0.282; 0.565]	0.181	0.218	[−0.247; 0.608]
Drug use	−0.656	0.642	[−1.917; 0.606]	−0.359	0.531	[−0.683; 1.402]	**0.675****	0.220	[0.243; 1.107]	**0.667****	0.222	[0.231; 1.104]
	R^2=0.18; $F_{10; 612}$= 12.95; p < 0.001			R^2=0.16; $F_{10; 612}$= 11.60; p < 0.001			R^2=0.17; $F_{12; 610}$= 11.03; p < 0.001			R^2=0.16; $F_{10; 612}$= 11.33; p < 0.001		

Note; *p < 0.05; ** p < 0.01; *** p < 0.001.
Coding systems: gender: 1= male 2= female. Occupation: 0= Unemployed; 1= student; 2= employed. Educational level= 1 = 8 years: 2= > 8 years < 13 years; 2= > 13 years.
Marital status: 1= unmarried 2= married or living with partner. Tobacco use= 0= no 1= yes. CAGE ≥ 2: 0= no 1= yes. Drug use: 0= no 1= yes.
Abbreviation: BSMAS= Bergen Social Media Addiction Scale; IGDS-SF= Internet Gaming Disorder Scale–Short-Form; MZQ= Mentalization questionnaire; BMI= Body Mass Index; CAGE= Cut-Annoyed-Guilty-Eye (CAGE) questionnaire; BSI= Brief Symptom Inventory.
